# Generating Input Data for Microstructure Modelling: A Deep Learning Approach Using Generative Adversarial Networks

**DOI:** 10.3390/ma13194236

**Published:** 2020-09-23

**Authors:** Felix Pütz, Manuel Henrich, Niklas Fehlemann, Andreas Roth, Sebastian Münstermann

**Affiliations:** Integrity of Materials and Structures, RWTH Aachen University, 52062 Aachen, Germany; Manuel.henrich@iehk.rwth-aachen.de (M.H.); Niklas.Fehlemann@rwth-aachen.de (N.F.); andreas.roth@rwth-aachen.de (A.R.); sebastian.muenstermann@iehk.rwth-aachen.de (S.M.)

**Keywords:** microstructure modelling, representative volume elements, dp-steel, machine learning, deep learning, wasserstein gan

## Abstract

For the generation of representative volume elements a statistical description of the relevant parameters is necessary. These parameters usually describe the geometric structure of a single grain. Commonly, parameters like area, aspect ratio, and slope of the grain axis relative to the rolling direction are applied. However, usually simple distribution functions like log normal or gamma distribution are used. Yet, these do not take the interdependencies between the microstructural parameters into account. To fully describe any metallic microstructure though, these interdependencies between the singular parameters need to be accounted for. To accomplish this representation, a machine learning approach was applied in this study. By implementing a Wasserstein generative adversarial network, the distribution, as well as the interdependencies could accurately be described. A validation scheme was applied to verify the excellent match between microstructure input data and synthetically generated output data.

## 1. Introduction

For modern applications, the microstructures and properties of steels have become exceedingly complex, utilising multiple phases and alloying concepts to improve mechanical properties for the specific use case. Even more basic materials, like dual-phase (DP) steels show complex correlations between their microstructure and mechanical properties, as well as their damage behaviour. The damage initiation and accumulation characteristics are especially important for the materials application in forming processes [1]. Here the damage introduced into the microstructure during forming will have a major influence on the components properties [2]. For dual phase steels the differences in strength between the two phases—ferrite and martensite—leads to strain partitioning in the microstructure, where the local microstructural neighbourhood is the key component for strain heterogeneity [3]. Thus, strain accumulates in ferrite, leading to specific damage behaviour for DP steels. Additionally, the morphology of a given dual-phase microstructure plays an important role in damage initiation and accumulation [4]. Multiple parameters of the microstructure have been identified that each play an important role, like grain size, martensite content, or grain shape. However, it is a very complex task to separate the multiple microstructural influences experimentally, since multiple parameters get changed when experimentally designing a new microstructure. To individually investigate each of the possible influencing factors, a numerical approach is more useful and commonly applied. The method of choice in that regard is microstructure modelling, which has been a key research focus in recent years and is continually developing. Commonly, two different methods are applied: Modelling the real microstructure and representing the microstructure by statistical distributions. For the first approach, the real microstructure is modelled based on microstructural analysis. For that procedure, mostly scanning electron microscopy is used to gather the required information in a suitable resolution [5]. The second option requires a statistical analysis of the base material. The acquired statistical information is then used to create small volume elements that show a good statistical representation of the microstructure [6]. A key requirement for these volume elements is that they need to contain enough information of the microstructure, while also being small enough to be clearly differentiated from the macroscopic structural dimension [7]. Due to this requirement, it is necessary to create statistical descriptions of the microstructure to reasonably represent it. To create a 2D representative volume element (RVE), the required data can be acquired from pictures of the microstructure, either via light optical microscopy (LOM) or scanning electron microscopy (SEM). Since the parameters of interest are on the order of magnitude of individual grains, SEM is used commonly. Here, multiple different detectors can be applied, while the electron backscatter diffraction (EBSD) detector delivers more in-depth information on each grain. The grain parameters that are most commonly applied in the creation of RVEs are the grain size, its shape (elongation, represented by the aspect ratio of the grain), and the slope of the grain describing the angle between the main axis of the grain and the x-axis of the picture (or the rolling direction). For three dimensional RVE these parameters need to be determined in all three directions in space. This can be done by serial sectioning, where the sample rotates inside a strong radiation beam and is scanned slice by slice, until a projection of the material can be acquired [8]. This method is, however, a very complex and time-consuming task. Thus, SEM or EBSD pictures from all three directions in space are often taken to create statistical distribution functions of the desired parameters depending on the respective spatial direction.

For the generation of the RVE model, the statistical distribution of the microstructure needs to be translated from the material to input parameters. For that reason, the parameters are commonly described by distribution functions that follow a log-normal distribution or a gamma distribution. Most often, histograms are used to fit the distribution functions to [6,9,10]. It has to be mentioned though that histograms are very dependent on bin size. Thus the resulting distribution function is also dependent on the bin size. Therefore, Kernel Density Estimation (KDE) should be the prevalent method, since they deliver robust values, where every data point is weighted by a function depending on the Kernel chosen (e.g., Gaussian) [11]. For the damage behaviour of a material grain, shape and size both play a very important role, thus modern RVE generation algorithms take many parameters into account that describe the grain shape, like slope or elongation [12,13,14,15]. The input for these parameters are separate, independent distribution functions of the applied parameters. Most materials, however, show some kind of interdependence of the parameters among each other. Thus, this study investigated the interdependencies of the microstructural parameters for steel DP800 with the goal to generate input data for any RVE generator that depicts the relations between the relevant parameters.

To describe these connections reliably, some kind of pattern recognition has to be applied to the input data of the EBSD pictures. Machine learning and deep learning methods are particularly suitable for this purpose, as they are able to reproduce the individual distributions of parameters and the relationships between them. They are often used for this purpose, in different areas of industry and science. Examples include the process industry [16], finance[17], the automotive sector[18], as well as research in the field of materials science [19]. Since machine learning has exactly the strengths needed to generate interdependent input data, a deep learning approach was used for this material. This allowed the generation of a virtually unlimited amount of input data, which follows the distribution functions of the individual parameters, but also exactly reflects the dependencies between individual parameters.

## 2. Analysis of the Input Data from the Real Microstructure

**Figure 1:**
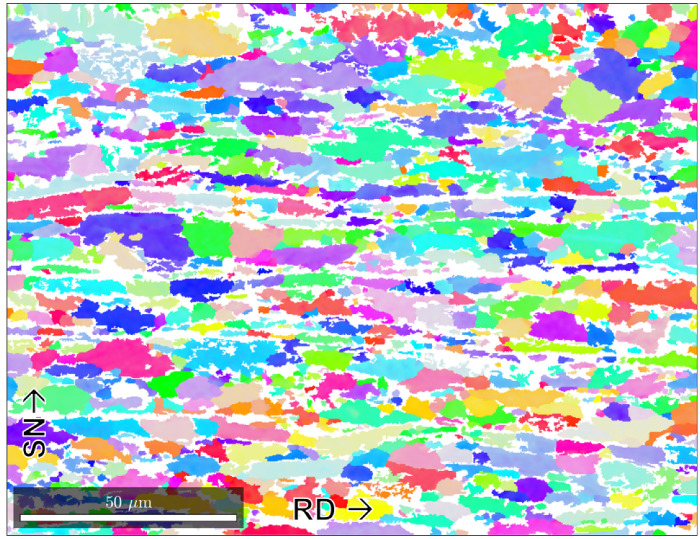
The electron backscatter diffraction (EBSD) of steel DP800 where the x-axis is the rolling direction (RD) and the y-axis is the sheet normal (SN) [20].

For the generation of statistically matching RVE, representing a specific microstructure, the analysis of the input parameters is a key factor. Usually, distribution functions of the input parameters are created and used to generate a discrete amount of input parameters for microstructure modelling. For the present study, a dual-phase steel (DP800) was utilised as the base material, since multiple important parameters are inherent to the microstructure that need to be characterised and represented by the RVE input data. In Figure 1, the microstructure of the DP800 steel is depicted as an EBSD picture. The different colours represent the different crystallographic orientations of ferrite, while martensite appears in white.

In this study only one direction in space was chosen for the analysis, since strong differences between different directions might apply, which increases the difficulty and complexity for the appropriate fitting of the input data. The rolling direction (RD)—sheet normal (SN) plane was chosen, since it shows the elongation of the grains and therefore more microstructural parameters that need to be present in the RVE.

**Figure 2:**
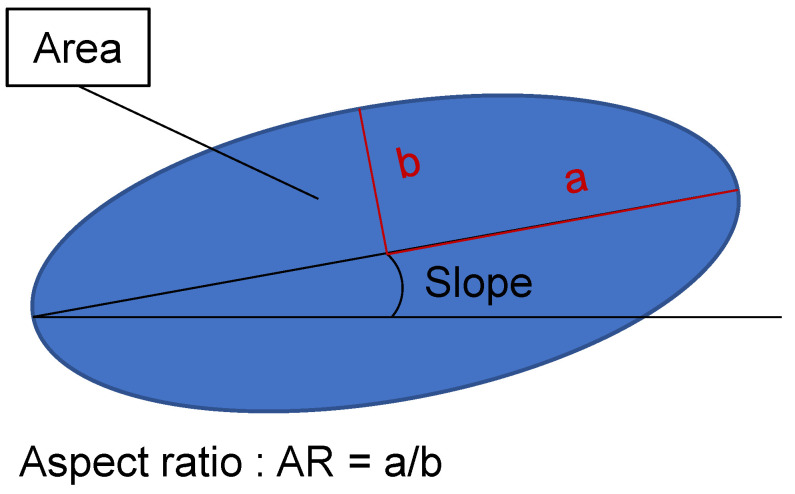
Schematic representation of a single grain with the associated shape parameters.

From this picture it can already be seen that multiple different parameters are inherent to ferrite in the microstructure. Since the EBSD pictures yield no specific data for martensite, as it can not be indexed, the focus for this study was put on ferrite, where multiple parameters are characterised by the EBSD evaluation. For any kind of microstructure, modelling geometric parameters of the grains are needed as input. In Figure 2, the geometric parameters of each individual grain are depicted.

All of the above mentioned parameters are obtained by using the MATLAB (Matlab R2019a—The Mathworks Inc., Natick, MA, USA) toolbox MTEX (MTEX version 5.3.0) [21]. This toolbox is able to analyse a lot of different characteristics of the microstructure, chief among which are the grain size, the aspect ratio (AR), and the slope, as well as the possible crystallographic orientation. Probably the most important aspect is the grains area, since the grain size also strongly influences mechanical properties. Apart form the size, the slope is an important factor and describes the tilt of a grain towards the x-axis of the EBSD picture taken, in this case the rolling direction (RD), where angles close to 0${}^{\circ }$ or 180${}^{\circ }$ means an elongation of the grain along the rolling direction. The last parameter is the AR, the ratio of the two half-axis *a* and *b*. A high AR stands for a bigger elongation, while an AR of 1 describes a circle. Both, the AR and slope influence the strain and stress concentration and distribution in the microstructure and are thus important to be depicted in the RVE. Apart from these geometrical factors, there are further material specific characteristics. The first is the grain orientation this is especially important when a texture is present in the material. Next are the different phases, in the case of DP800, ferrite and martensite. Additionally neighbourhood relations between the grains might be taken into account. All of the parameters mentioned above can be gathered from the data generated by the EBSD measurement. From this measurement, a list of grains and their corresponding characteristics can be obtained that is afterwards used for further analysis.

**Figure 3:**
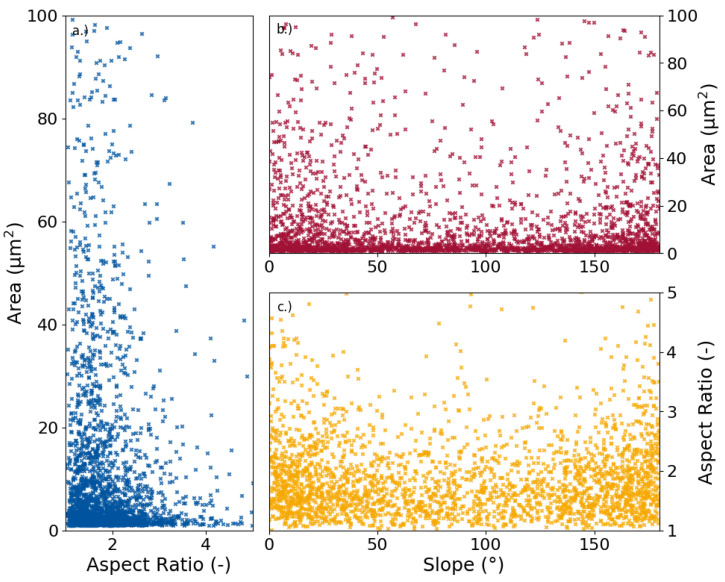
Scatter plots of the dependencies between the three main grain parameters, grain size and aspect ratio (**a**), grain size and slope (**b**), and aspect ratio and slope (**c**).

For the sake of simplicity, the focus was put on ferrite in this study as mentioned before. The geometrical factors of the grain were chosen for a deeper analysis, while the grain orientation was not taken into account, since no strong texture is present in the investigated material. For most RVE generators, the geometrical parameters (grain size, elongation, and slope) are commonly implemented into the statistically-generated microstructure model through singular distribution functions of the separate parameters. From these, separate sets of input is generated for each parameter that are not interconnected. However, when the parameters are plotted against each other as two-dimensional scatter plots where each dot represents a unique grain, certain trends can be observed between the parameters Figure 3.

The scatter plots show the connectivity between the main parameters. In Figure 3a the grain size against the aspect ratio is depicted. Most grains are concentrated in the bottom left corner meaning they posses a relatively small area and aspect ratio. However, the most noticeable property is the fact that grains above 20 μm^2^ grain size show a tendency towards smaller aspect ratios of 2.5 or lower, while for the smaller grains, higher aspect ratios become increasingly likely. Another strong connection is visible in Figure 3c. Here it is identifiable that more grains have a slope around $0^{\circ }$ or $180^{\circ }$ respectively, which both go along the x-axis (RD). Especially notable is the fact that towards a slope of $90^{\circ }$, which is perpendicular to the RD, a significantly smaller quantity of grains show aspect ratios above 2.5. This means, that grains oriented perpendicular to the rolling direction are not as elongated as the grains that go along it. The same can be said to lesser extent for Figure 3b. For a comparison of area and slope a concentration at $0^{\circ }$ or $180^{\circ }$ is notable, as is the smaller quantity of grains with a bigger area perpendicular to the rolling direction. A few dependencies can therefore be identified: Bigger grains show comparable smaller aspect ratios. They are also less likely to be oriented perpendicular to the rolling direction. Additionally, grains that have a larger aspect ratio tend to have a slope around $0^{\circ }$ or $180^{\circ }$ respectively.

This, however, leads to some conclusions in regards to the way the microstructure model needs to be created. Separate distribution functions that provide input data for the creation are not suitable since that way the interdependencies observed above are not taken into account. If all parameters are generated individually, it is possible to create a grain with a large grain size over 50 μm, a high AR near 5, and a slope value near $90^{\circ }$. This theoretical grain does not exist in the real microstructure. Thus, a solution for the generation of input data is needed that takes the interdependencies of the parameters into account.

## 3. Machine Learning Networks (MLN)

**Figure 4:**
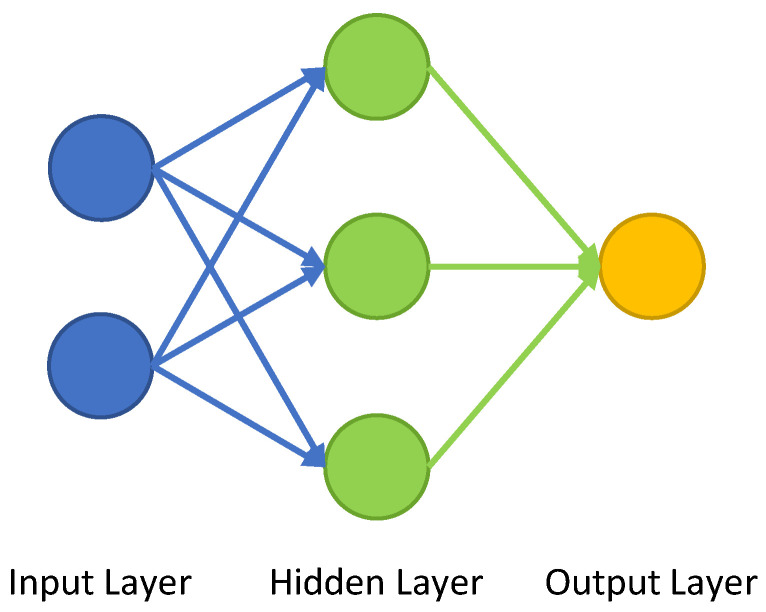
Schematic representation of a machine learning algorithm (MLA) showing a different layer, after [23].

For any problem that revolves around understanding core similarities of any given data set, machine learning (ML) is an ideal approach. Machine learning algorithms (MLA) are able to learn dependencies in data or even images, that would be hard to grasp for the human applicant. They especially thrive, given multidimensional data frames, that show a certain degree of interconnectivity or interdependency. For MLA, the deep learning method roughly replicates the operating principle of the human brain. Therefore, these neural networks (NN) consist of a number of neurons that are structured in layers to reinforce learning procedures. Usually these layers consist of an input layer and a number of hidden layers, one of which is the output. The schematic structure of a NN is depicted in Figure 4. Here, from each of the inputs, a synapse is leading to the first hidden layer. For each of the inputs, a weight and a bias is given to the input values. At the subsequent neuron, in the hidden layer, the incoming values are summed up and an activation function is applied to the weighted sum. The output of the neuron is the input with the applied activation function, multiplied with a new weight. This new value is then again used for the next layer, which would be the output layer in case of the schematic representation visible in Figure 4. After the output is created, a back propagation takes places where the weights of the neurons are updated to improve the NN quality [22].

The activation function is one of the important parameters that need to be chosen carefully for the MLA, while the weights of the neurons, as well as the bias are the training parameters of the NN, that are iteratively fitted during the learning process. The number of hidden layers in a NN are called the depth, while the number of neurons in each layer is called the width.

The machine learning network that is to be applied in this study has to be able to represent a distribution of different parameters, as well as their interdependencies. For the description of any statistical distribution, unsupervised machine learning is best suited. For generative machine learning methods, generative adversarial networks (GAN) are quasi state of the art. They are especially useful to reproduce the statistical distribution of a set of parameters, as well as their interdependencies, be it information from images or raw text-based data [24]. The training of GANs, however, is well known for being unstable and delicate with the main problems being mode collapse, non-convergence, and diminished gradient [25].

The principle of the GAN is that it pits two NNs against each other. The two NNs are a generator and discriminator. The generator generates data trying to reproduce the input data as well as possible. The key mechanism for GANs is the adversary of the generator network, the discriminator. This NN learns to distinguish between generated and input data. The competition between the two networks leads to a distinct improvement in the results during the training epochs.

The instability issues of the GAN model stem from the applied loss function. A loss function in neural networks reduces all aspects of a complex system whether good or bad to a single scalar number that allows for a ranking and comparison of solutions. Originally, the GAN model used a minmax loss function, where the generator tries to minimise the target function, while the discriminator maximises it. To get rid of the major instabilities of the first GAN model, Arjovski et al. [26] changed the loss function of the GAN model to the Wasserstein metric. This change leads to an improved stability of the network during training, while still retaining an excellent capability to describe the parameters distribution and their interdependencies. The advantage of the Wasserstein implementation is that the generator can still learn, even if the discriminator is well trained, in addition to no observable mode collapse.

## 4. Training of the MLA

To gather input data for the training of the machine learning networks, EBSD pictures of the microstructure along the rolling direction of the thickness of the steel sheet were taken at the Institute for Physical Metallurgy and Materials Physics of RWTH Aachen University. Only one direction in space was used for the MLA, since the differences between the directions are quite significant. With these pictures, a list of the grains in the section of the material can be obtained. Since MLN requires a large amount of input data, a large area was measured in the same direction that is indicated in Figure 1. In this way, slightly more than 3000 grains were captured to be used as input for training. As mentioned above, the geometric grain data of ferrite were chosen as training data. Additionally, the mean misorientation angle was used as a mean to validate the results further. This angle defines the average deviation of the orientation inside a grain and is used to define grain boundaries for EBSD pictures.

**Figure 5:**
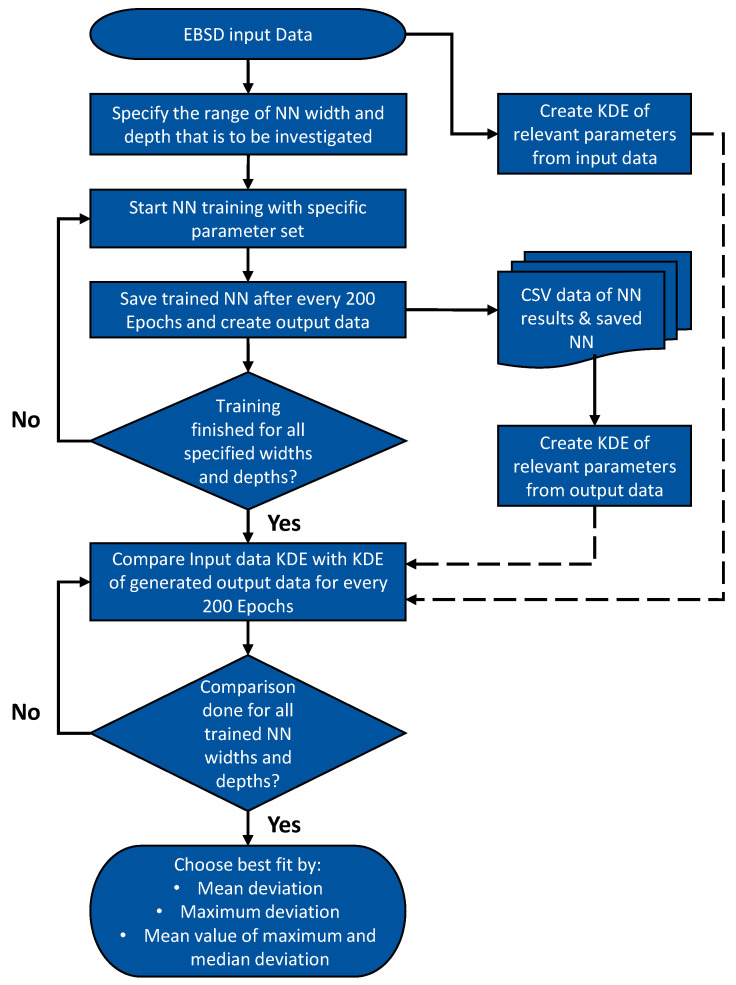
Flow chart of the training approach and calibration regimen applied to find the best fit parameters for the generation of synthetic microstructure data.

An implementation of the Wasserstein GAN (WGAN) algorithm was applied as the chosen algorithm and an effort was made to change the network to be able to run on a GPU-type Tesla P100, which decreases the training time required quite significantly. For the implementation of the WGAN scheme, two feedforward neural networks were applied: One as the discriminator and one as the generator. For the activation functions a ReLu function was used for all but the Output layer of the discriminator, which uses a Sigmoid function, since the result of this layer has to be a probability and Sigmoid functions give results between 0 and 1 [24]. For all of these implementations, the Pytorch library (version 1.5) was used [27]. The approach that was used to find the best possible NN for the input data is described in Figure 5 as a flow chart and in more detail in the text below.

Neural networks require hyperparameters to be fully functional. These are a set of parameters that are defined for the NN before the training starts. The most important ones are the width and depth of the network, as explained above. These two hyperparameters are key for the training process, since they determine the number of neurons and the depth of the network. For each neuron, a weight exists as was explained above. By adapting the weights of the neurons for each processed batch the network actually learns the target distribution. Therefore, the number of neurons in a layer, as well as the amount of layers contributes immensely to the quality of the trained NN. Thus, width and depth were iteratively fitted in this study. This was done by training multiple NN with varying hyperparameter sets (especially for width and depth), which were changed iteratively in a looped approach. Therefore the parameter sets to be tested were defined before the training and then subsequently tested.

To train the MLA, a mini batch gradient descent procedure was utilised. In this procedure, the training data set is split into many, randomly-generated sub sets. The algorithm processes each mini batch separately and compares the cost function in relation to the current mini batch data. Subsequently, the network parameters are updated accordingly. This is done iterating over all mini batches, until the whole data set has been evaluated. This complete process is called an epoch. For the training in this study, a batch size of 64 was used, which is in line with batch sizes recommended in the literature [28,29].

Other hyperparameters usually describe the learning rate of both the generator network and the discriminator network. However, the learning rate was automatically adapted, since an optimisation algorithm called RMSProp was used for the back propagation. This optimisation approach requires a low learning rate which is inherent to WGAN networks. Thus the influence of these parameters is negligible. Two important parameters are the clipping value and the dropout. Both variables represent values that are important in the NN to avoid overfitting and underfitting [30,31].

When the framework is defined, the training of the NN begins. Since the NN changes slightly after every epoch, it is important to create intermediate snapshots of the NN, as well as the output data in the form of a CSV file after a defined number of epochs, 200 in the case of this study. Additionally, only a defined number of epochs should be trained, since MLA are prone to overfitting in higher epochs.

After the training of all the different NN for multiple parameter sets is complete, the best fit needs to be found. To do so, a script was written, which creates a KDE of every microstructural parameter taken into account for the MLA training. Additionally, the KDE of every taken snapshot are created of the same parameters, since this is the output of the generator network. Subsequently the fit between the input KDE and synthetic data KDE are evaluated. This is done by investigating three types of deviation between the KDE of the real and synthetic data: The mean deviation, maximum deviation, and mean value of both mean and maximum deviation. All three are returned and can be checked by the user. Ideally, a NN snapshot can be chosen that shows the smallest deviation for all three values in comparison to the rest of the NN.

## 5. MLA Results

**Figure 6:**
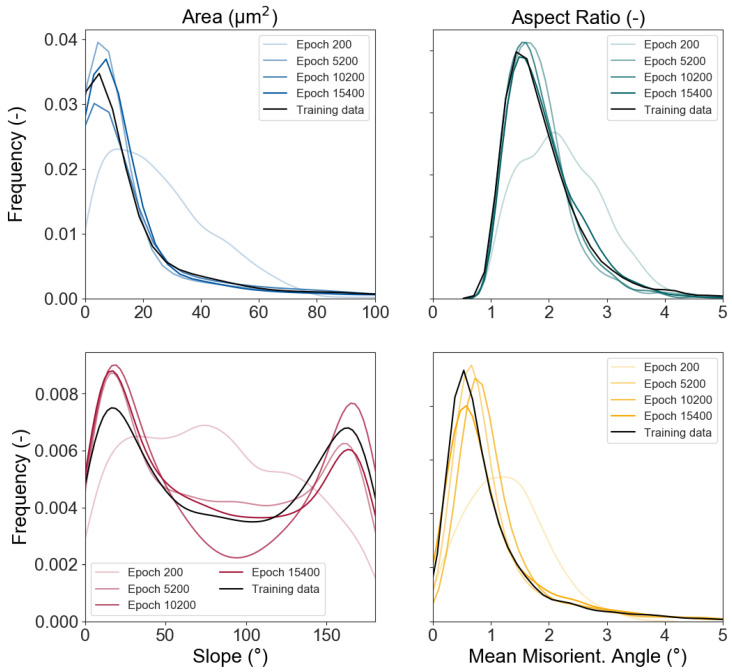
Development of the output of the neural networks (NN) over multiple epochs, compared to the input data. Epoch 15,400 is the best fit for the applied input values.

After the training of the NN is completed, the network with the best results can be chosen. For this, the deviation of the KDE are utilised as described before. Since they show a significant development over the epochs, the best fit is the best averaged value, where most KDE curves fit the input KDE very well. The comparison of the best fit at epoch 15,400 with the input data, as well as former epochs can be seen in Figure 6.

**Figure 7:**
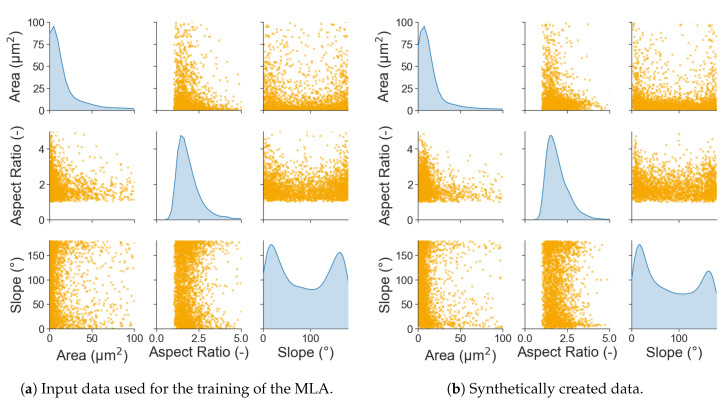
Comparison between the input data used for the training of the MLA and the output of the generator network at its best fit epoch. Dependencies between the different microstructural parameters shown for a better evaluation of the results.

Here, a significant improvement of the fit is visible, where epoch 200 is an unoptimised guess, while the network improves over time with the training and is able to represent the distributions of the input parameters at the best fit epoch accurately. From the trained generator, network data can then be extracted, which is usable as input for e.g., RVE creation. The output of the NN follows the same format of the input. This means that every parameter contained in the input data will be present in the output created by the MLA. Since the focus is the creation of input data that geometrically represents a grain (Figure 2), the main features to compare are area, aspect ration, and slope. In Figure 7, pairplots of both the input data and synthetically generated output data are presented. On the diagonal, the KDE distributions are shown, while the other graphs show the respective parameters plotted against each other. From these pictures the compliance between real and synthetic grain data appears to be excellent.

**Figure 8:**
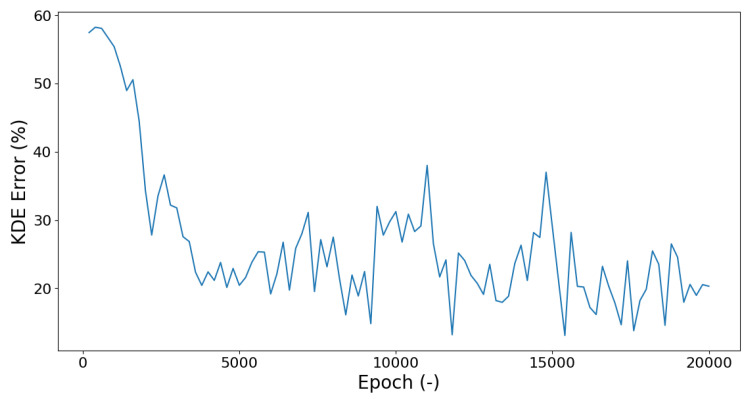
Development of the error of the output Kernel Density Estimation (KDE) compared to the input KDE over the course of training.

As mentioned before, the best fit of the MLA and the input data is determined by a comparison of the KDE for each investigated parameter. The course of this error margin between input and output over the epochs is depicted in Figure 8. For this figure the mean deviation between the input and output KDE were calculated. The deviation was calculated in percent after the following formula:

(1) $$KDE\;Error=100\cdot \frac{1}{n}\cdot \frac{\sum |\bar{x}-x|}{\bar{x}}$$

where $\bar{x}$ is the input data KDE and *x* is the artificial output data KDE. Due to this analysis, the lowest deviation between the KDE can be determined. In this case, the best fit was determined to be Epoch 15,400 which is the lowest point in the curve with an error margin of about 5%.

## 6. Validation of the MLA Results

**Figure 9:**
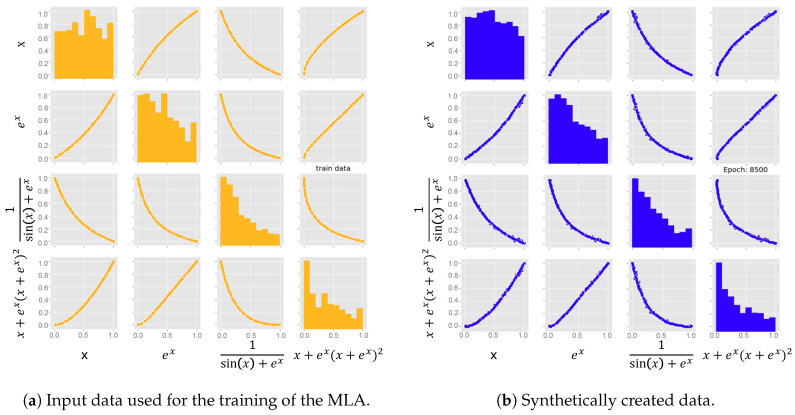
Validation to see, whether the MLA is capable of representing the dependencies of its input data set by applying multiple different interdependent equations.

By checking the KDE error of the singular plots it is not sufficiently validated if the NN can fully describe the microstructure. Since the most important task of the machine learning algorithm was to represent the interdependencies of the microstructural parameters, a study was conducted to investigate whether the applied algorithm is capable of handling interdependencies between input data. Therefore the MLA was trained on a specifically created data set with four parameters:

(2) $$\begin{array}{cc} f_{1}\left(x\right)= & x \end{array}$$

(3) $$\begin{array}{cc} f_{2}\left(x\right)= & e^{x} \end{array}$$

(4) $$\begin{array}{cc} f_{3}\left(x\right)= & \frac{1}{\sin\left(x\right)+e^{x}} \end{array}$$

(5) $$\begin{array}{cc} f_{4}\left(x\right)= & e^{x}\cdot {\left(x+e^{x}\right)}^{2}+x \end{array}$$

where *x* was generated from a uniform distribution. The other functions were arbitrarily chosen, with the criteria being that they all need to be dependent amongst each other. In this case Equation (2) is dependent on Equation (1), while (3) and (4) are dependent on both, (1) and (2). Thus all values were interconnected and the implemented MLA was trained on this data set. The results presented in Figure 9 show that the MLA is able to reproduce the input data very accurately. Both the distribution of each specific equation and the interdependencies as well show no significant deviation. It can therefore be assumed that the implemented MLA is capable of representing any interdependencies that are present in the input data and it can thus be assumed that the dependencies of the microstructure are represented by the MLA.

**Figure 10:**
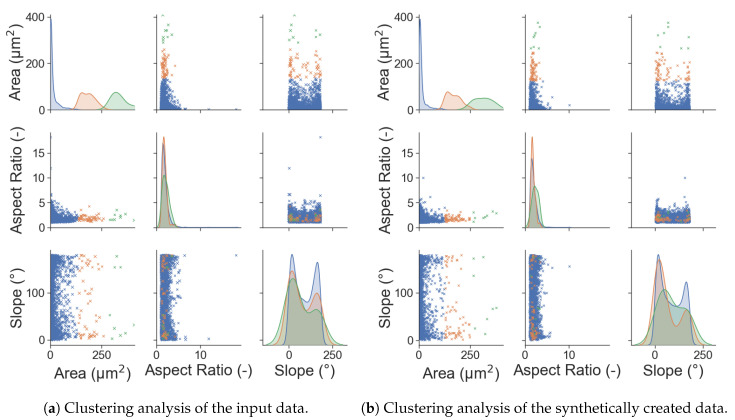
Comparison between the clustering analysis of the input data and the generated output data. The area was divided into three equally sized sections for this analysis.

To further investigate whether the dependencies between the microstructural parameters were accurately reproduced, a clustering analysis was performed on the microstructure data. For this analysis, the area data were divided in three evenly sized batches that were subsequently plotted like the results were before. To see if the dependencies were correctly reproduced, each of the batches was given a different colour, allowing individual batches to be tracked. This clustering analysis is pictured in Figure 10. In this figure, the orange and green points are of special interest. The KDE distributions fit very accurately, which was to be expected after the very good fit of the complete KDE functions before. A comparison of the AR—Slope chart shows that each of the orange and green points (which stand for grains with larger areas) is in a very similar position, but not the same. Most of the relevant points are at smaller aspect ratios and positioned near the 0${}^{\circ }$ or 180${}^{\circ }$. No outliers are found in this analysis, which leads to the conclusion that the interdependencies of the input data can be accurately portrayed.

## 7. Conclusions

This study presented a solution on how to generate input for microstructure modelling that is true to the real microstructure. Commonly, singular distribution functions are applied to describe the input for the microstructure model. However this study showed that an approach like that is not suitable to describe any given microstructure in detail. Since there are at least three relevant parameters to each grain for each direction in space (area, aspect ratio, and slope) all of these have to be represented accurately by statistic descriptions. The three aforementioned parameters are, in fact, all dependent on each other, where a relatively large grain tends to have smaller aspect ratios as well as a slope more parallel to the rolling direction.

To solve this challenge, this study applied a WGAN machine learning network to generate input data that represents all of the interdependencies. The results show that the NN learned the distribution functions of the singular parameters very well. The generated output resembled the input quite accurately, while also representing the dependencies between the parameters. To validate these findings, a study was carried out to see whether the implemented NN was able to recreate numerical relations between the different parameters. It was found that the WGAN algorithm was capable of recreating the equations. To validate the results regarding the microstructural features, a clustering analysis showed that the output data resembled the input data very accurately.

The error of MLN when comparing input and output KDE oscillated quite strongly. In Figure 8, an initial significant improvement could be observed after which the curve started to spike around a median value. It could therefore be assumed that the training required for this network could be significantly shortened, since a comparably good result was achieved after about 9000 Epochs. Thus, the time needed for the training of each network could be reduced if the amount of Epochs for a full training were lowered.

The implemented network in this study was trained on just one direction in space (rolling direction x sheet normal). To precisely describe any microstructure, however, it is necessary to show the influences of all three directions. Here the approach can be expanded. However, it is not an easy feat to generate statistically relevant information of the three dimensional structure from two dimensional pictures. A possible solution would be to simply train three networks, the question that remains unanswered is how the individual parameters of the three spatial directions relate. Answering this question will be a key focus in future work.

In comparison to other machine learning applications, this network was trained on a relatively small sample size. This was done deliberately, since in everyday research it is not always feasible to create a magnitude of EBSD pictures. The results from this study show therefore that the applied concept works very accurately even on small sample sizes, where bigger data sets could only improve the quality of the output. Thus, this approach is suitable to be implemented even for small sample sizes and projects with smaller amounts of time and resources dedicated. However, while the WGAN model can be trained on relatively small data sets, it is important to use enough data. If a very low number of grains (e.g., 200) is used as the input, the resulting data is no longer statistically relevant to the target microstructure. Thus, a sample set has to be applied that is representative of the real microstructure.

While the error of the MLA was remarkably low at 5%, there were some bigger deviations when comparing the input and output KDE. To improve this deviation, multiple approaches are possible. First of all the precision of the ML model increased with the addition of more input data. Therefore, a more exact representation of the microstructure could be achieved if the input data was increased likewise. Another option could be to apply a different type of optimisation, where the Wasserstein loss is used as the comparison of real to synthetic data. This approach appears to be promising and is currently developed.

For this study, DP steel was chosen as the input microstructure. Since the manufacturing process for DP steels incorporates multiple steps, like hot rolling, cold rolling, and coating, they often show a complex microstructure where even the ferrite phase has multiple characteristics that need to be considered to accurately represent the material. However, the focus of this study was put on ferrite, since the description of martensite cannot be achieved by simple EBSD analysis, as mentioned above. Additionally, for the present material, martensite exists in bands as well as singular islands. A differentiation between those two is necessary for a thorough analysis and an automated band detection algorithm is currently being worked on.

To apply the synthetically generated microstructure, an RVE generation algorithm is needed, like the one developed by Henrich et al. [12]. The combination of these two approaches with a complete characterization of the martensite phase will lead to a very close representation of the actual microstructure. Additionally, an extension of the applied ML model is quite easily done, as it only requires adding more columns to the input of the ML model. Thus, the crystallographic orientations of the microstructure can be incorporated and represented in the resulting RVE. From that microstructure model, mechanical properties of the phases can be concluded and in depth studies of the response of the different phases to mechanical loading can be undertaken.
